# Nervonic acid confers neuroprotection in a zebrafish model of diabetic neuropathy by promoting myelin repair and metabolic modulation

**DOI:** 10.3389/fphar.2026.1721486

**Published:** 2026-02-18

**Authors:** Huan Ge, Mingzhu Dai, Qinwen Wang, Bingbing Cao, Ximin Zhu, Jie Guo, Tao Zhang, Shasha Wang, Yiqiao Xu, Lushan Yu

**Affiliations:** 1 College of Pharmaceutical Sciences, Zhejiang University, Hangzhou, China; 2 Hunter BioInsight Technology, Inc., Hangzhou, China; 3 Hunter Biotechnology, Inc., Hangzhou, China; 4 Zhejiang Provincial Engineering Research Center for Innovative Small-Molecule Drugs, Jinhua, China

**Keywords:** diabetes neuropathy, metabolomics, myelin repair, nervonic acid, neuroprotection, oxidative stress

## Abstract

**Introduction:**

Diabetic neuropathy (DN) is one of the most common and debilitating complications of type 2 diabetes mellitus (T2DM), yet effective therapeutic strategies remain limited. Nervonic acid (NA) is recognized for its neuroprotective and anti-inflammatory properties. However, its role in DN has not been fully elucidated. In this study, we investigated the protective effects of NA against T2DM-induced DN using a zebrafish model and explored the underlying molecular mechanisms.

**Methods:**

T2DM was induced in zebrafish larvae through a high-fat, high-glucose diet combined with a low dose of streptozotocin. Larvae were subsequently treated with NA at concentrations of 125, 250, or 500 μg/mL. Motor function, myelin integrity, neutrophil infiltration, and reactive oxygen species (ROS) levels were evaluated using fluorescence imaging and histological staining. Gene expression analysis was performed by quantitative real-time PCR. Metabolomics coupled with KEGG enrichment analysis was applied to identify NA-regulated metabolic pathways.

**Results:**

NA significantly preserved myelin integrity, reduced neutrophil infiltration, and lowered ROS levels in DN zebrafish. Expression of myelin-related genes (*mbpa* and *mpz*) was upregulated, while pro-inflammatory cytokines were downregulated following NA treatment. Metabolomic profiling revealed that NA reversed diabetes-associated dysregulation in purine metabolism, energy metabolism, vitamin B6 pathways, and redox homeostasis. Key metabolites including guanosine monophosphate, adenosine triphosphate, pyridoxal 5′-phosphate, and L-glutathione were markedly restored toward normal levels.

**Discussion:**

These findings demonstrate that NA confers robust neuroprotection in DN by alleviating inflammation and oxidative stress, preserving neuronal structure and function, and reprogramming key metabolic pathways.

## 1 Introduction

Diabetes mellitus (DM) is a chronic and complex metabolic disorder characterized by persistent hyperglycemia due to either impaired insulin secretion or defective insulin action (Niu et al., 2024). The two most prevalent subtypes are type 1 diabetes mellitus, caused by autoimmune destruction of pancreatic β-cells, and type 2 diabetes mellitus (T2DM), primarily associated with insulin resistance and relative insulin deficiency (Antar et al., 2023). Regardless of etiology, prolonged hyperglycemia induces widespread pathological consequences, including cardiovascular complications, retinopathy, nephropathy, impaired wound healing, and a spectrum of neuropathies (Paul et al., 2020). Among these, diabetic neuropathy (DN) is one of the most common and debilitating complications, affecting approximately 50% of diabetic patients. Clinically, DN is characterized by sensory deficits, such as tingling, numbness, and allodynia, along with motor impairments, including weakness and loss of coordination (Callaghan et al., 2012). Current therapeutic strategies primarily focus on glycemic control and symptomatic pain relief, with limited efficacy in halting or reversing nerve damage. Hence, there is an urgent need to explore novel therapeutic interventions targeting the multifactorial pathogenesis of DN.

The mechanisms underlying DN are multifaceted, with chronic hyperglycemia playing a central role. One of the key pathological pathways involves the overproduction of reactive oxygen species (ROS), leading to oxidative stress, mitochondrial dysfunction, and cellular apoptosis in both neurons and glial cells (Cernea and Raz, 2021). In addition, T2DM is frequently associated with dyslipidemia, which contributes to neural lipotoxicity, endoneurial ischemia, and inflammation. Prolonged exposure to high glucose and abnormal lipid levels promotes the accumulation of advanced glycation end-products, activates the NF-κB signaling pathway, and upregulates pro-inflammatory cytokines such as TNF-α and IL-6. These events impair axonal transport, damage the blood-nerve barrier, and inhibit myelin regeneration, ultimately leading to progressive demyelination and axonal degeneration (Wang et al., 2020a; Lin et al., 2023). Notably, both clinical and experimental studies have shown that diabetic neuropathy can progress despite optimal glycemic control, indicating that factors beyond hyperglycemia, such as metabolic dysregulation and inflammatory stress, play critical roles in its pathogenesis (Yang et al., 2025; Arı et al., 2025). Consequently, therapeutic strategies targeting multiple pathological pathways, including oxidative stress, inflammation, and lipid metabolism, may provide more comprehensive and effective protection against DN.

In recent years, the zebrafish (*Danio rerio*) model has emerged as a valuable vertebrate system for studying metabolic diseases, including diabetes and its neurological complications (Lee et al., 2024). Owing to its genetic similarity to mammals, transparent embryos, and ease of manipulation, zebrafish enables high-throughput screening and real-time *in vivo* imaging of metabolic and neural changes (Qi et al., 2022). A zebrafish model of T2DM can be successfully established by a combination of high-fat high-glucose (HFHG) diet and low-dose of streptozotocin (STZ), which replicates features of insulin resistance, β-cell dysfunction, and persistent hyperglycemia (Cao et al., 2023). In this model, zebrafish larvae exhibit hyperglycemia-induced oxidative stress, impaired locomotor activity, abnormal myelination, and inflammation, which parallel the clinical features of DN in humans (Md Razip et al., 2022). Furthermore, the conserved structure of the peripheral nervous system and the presence of myelinated axons in zebrafish allow for the investigation of demyelination and axonal damage. As such, HFHG/STZ induced type 2 diabetes zebrafish model provides a powerful platform to explore the therapeutic efficacy of candidate compounds targeting diabetic neurodegeneration.

Nervonic acid (NA), a very-long-chain monounsaturated fatty acid (24:1, ω-9), is an essential structural component of sphingolipids and myelin in the central and peripheral nervous systems (Zheng et al., 2024). NA has garnered interest due to its potential in promoting remyelination, enhancing nerve regeneration, and improving cognitive function. Recent studies have demonstrated that NA exerts significant antioxidant and anti-inflammatory effects by modulating ROS scavenging enzymes and suppressing pro-inflammatory signaling pathways (Yuan et al., 2023; Liu et al., 2024). Additionally, NA plays a regulatory role in lipid metabolism by enhancing mitochondrial β-oxidation and reducing lipid accumulation, thereby alleviating lipotoxicity-induced neuronal stress (Destaillats et al., 2025). These multifaceted properties suggest that NA may protect against the neural damage associated with T2DM through mechanisms including myelin sheath repair, attenuation of oxidative stress, suppression of inflammation, and normalization of lipid metabolism. However, the neuroprotective effects of NA in the context of diabetic neuropathy, particularly in the zebrafish model, remain largely unexplored.

In this study, we established a zebrafish model of T2DM by combining a HFHG diet with STZ exposure. Using this model, we aimed to investigate the neuroprotective effects of NA against T2DM-induced DN and to explore its potential underlying mechanisms. Through this approach, we hope to provide novel insights into the therapeutic potential of NA as a multi-targeted strategy for preventing or ameliorating DN.

## 2 Materials and methods

### 2.1 Major reagents

NA (HY-N2526, MedChemExpress, NJ, USA) was dissolved in DMSO (D2650, Sigma-Aldrich, Missouri, USA) to prepare a 100 mg/mL stock solution, with the final DMSO concentration maintained below 0.1% (*v*/*v*). Glucose (G107850), STZ (S766968), anhydrous citric acid (T632817), and sodium citrate (S425417) were purchased from Shanghai Aladdin. Egg yolk powder was obtained from Zhejiang Aige Biotechnology. Pioglitazone tablets and acarbose (A832179) were purchased from Jiangsu Deyuan and Shanghai Macklin, respectively.

### 2.2 Zebrafish maintenance and embryo collection

This study utilized four zebrafish strains: the wild-type AB strain (WT) and three transgenic lines including Tg(*ins*:EGFP), Tg(*hb9*:EGFP), and Tg(*mpx*:EGFP), all sourced from Hunter Biotechnology Co., Ltd. (Hangzhou, China). All zebrafish were maintained at 28 °C ± 0.5 °C under a 14-h light/10-h dark photoperiod. Two pairs of adult zebrafish were placed in spawning tanks the night before fertilization. The next morning, spawning was induced by 30 min of light exposure. Healthy embryos were collected at 3 h post-fertilization and incubated in E3 medium at 28.5 °C under the same light and dark cycle. All experimental protocols were reviewed and approved by the Ethics Committees of Hunter Biotechnology Co., Ltd. (Approval Numbers: SYXK (Zhejiang) 2022–0004 and IACUC-2024–0115–01). Animal housing and care were conducted in accordance with AAALAC accreditation standards (Accreditation Number: 001458).

### 2.3 Establishment of the zebrafish T2DM model

**FIGURE 1:**
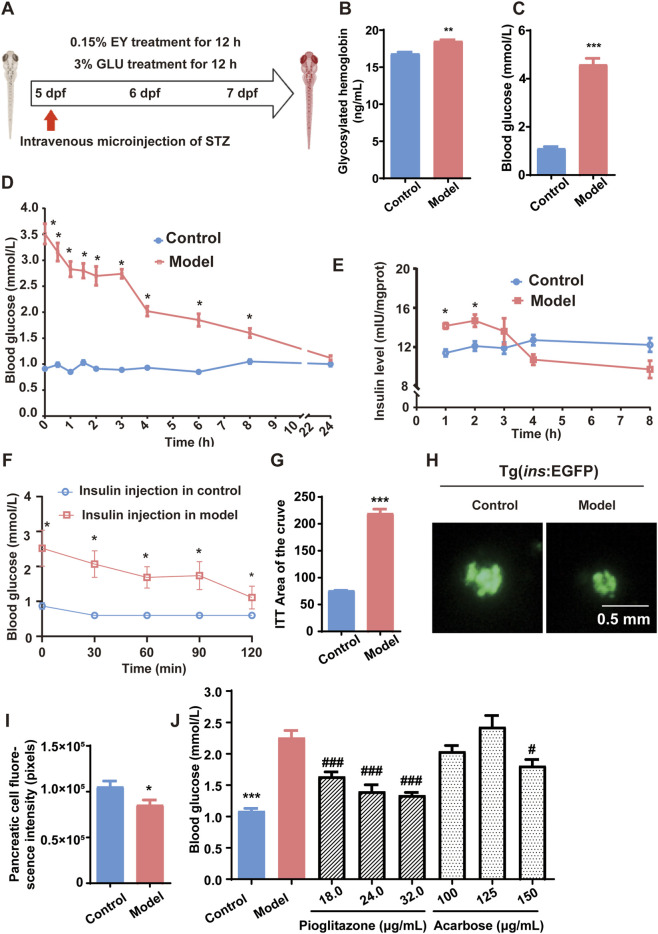
Establishment and validation of a zebrafish model of T2DM. **(A)** Schematic representation of the T2DM induction protocol in zebrafish. **(B)** HbA1c levels. **(C)** Blood glucose levels. **(D)** Blood glucose levels after withdrawal of HFHG feeding. **(E)** Insulin levels following dietary withdrawal. **(F)** Blood glucose response to exogenous insulin during the insulin tolerance test. **(G)** AUC analysis of glucose levels in the insulin tolerance test. **(H)** Fluorescence microscopy images of pancreatic β-cells in Tg(*ins*:EGFP) transgenic zebrafish. **(I)** Quantification of EGFP fluorescence intensity in pancreatic β-cells. **(J)** Blood glucose levels following treatment with varying concentrations of pioglitazone and acarbose. Data are presented as mean ± standard deviation (SD). *n* = 3 biological replicates for **(B)**, *n* = 10 biological replicates for **(C-J)**. Panels **(B–I)** were analyzed using Student’s t-test for two-group comparisons. Panel **(J)** was analyzed using one-way ANOVA followed by Tukey’s post-hoc test for multiple comparisons. *p < 0.05, **p < 0.01, ***p < 0.001 vs. control group; ^#^ *p* < 0.05, ^###^ *p* < 0.001 vs. model group.

To establish a zebrafish model of T2DM, WT zebrafish larvae at 5 days post-fertilization (dpf) were randomly selected and maintained in beakers, with 30 larvae in each 25 mL beaker. The model group was induced by intravenous injection of STZ. Before injection, the larvae were anesthetized in 0.05% tricaine solution for 2 min and then positioned ventral side up in grooved agarose molds. A glass capillary needle with a 20 μm tip diameter, connected to a microinjector, was used to administer 10 nL of STZ solution (50.0 mg/mL, prepared in 0.1 M citrate buffer, pH 4.4) to each larva. The STZ solution was freshly prepared and used within 30 min. The control group was injected with an equivalent volume of citrate buffer. Following injection, larvae were transferred to E3 medium and, after resuming normal swimming behavior, returned to the beakers. They were then subjected to a 48-h alternating HFHG feeding protocol: 0.15% egg yolk powder solution for 12 h during the day, followed by 3% glucose solution for 12 h overnight. This protocol was conducted from five to seven dpf, with slight modifications based on previous studies (Wang et al., 2020b; Kulkarni et al., 2022; Bentanachs et al., 2025), as illustrated in Figure 1A. Following successful model establishment, zebrafish were treated with varying concentrations of pioglitazone (18, 24, and 32 μg/mL) and acarbose (100, 125, and 150 μg/mL). Blood glucose levels were subsequently measured to evaluate the model’s stability and its pharmacological responsiveness.

### 2.4 Metabolic validation of the zebrafish diabetes model

To confirm the successful establishment of the zebrafish diabetes model, key metabolic indicators including blood glucose and glycated hemoglobin were measured. For blood glucose measurement, 10 zebrafish larvae were washed twice with E3 medium following treatment, then dried at 60 °C for 2 h. Each zebrafish was homogenized in 1 μL of ddH_2_O using a fully automated rapid sample grinder (JXFSTPRP-24L, Shanghai Jingxin Experimental Equipment Technology Co., China), followed by centrifugation at 2,500 rpm and 4 °C for 20 min, after which the supernatant was collected for subsequent analysis. Glucose levels were measured using a blood glucose meter (ACCU-CHEK Performa, Mannheim, Germany). For glycated hemoglobin, zebrafish were homogenized in 0.9% saline at a 1:9 (*w/w*) ratio, then centrifuged at 2,500 rpm and 4 °C for 20 min. Each experimental group included three biological replicates, with each replicate consisting of 30 zebrafish larvae. The resulting supernatant was used for subsequent assays. Protein concentration was determined using the bicinchoninic acid protein assay (BCA) method (Boster, Wuhan, China). Levels of glycated hemoglobin was quantified using zebrafish-specific ELISA kits (Enzyme-linked Biology, Shanghai, China) according to the manufacturers’ protocols.

### 2.5 NA administration

NA was administered to zebrafish embryos at concentrations of 125 μg/mL, 250 μg/mL, and 500 μg/mL. The NA stock solution was prepared by dissolving NA in DMSO, and the appropriate volumes of the stock solution were added directly to the E3 medium to achieve the desired final concentrations. Zebrafish embryos were exposed to NA in the medium starting at five dpf and maintained in the medium for the duration of the experiment. The control group was treated with an equivalent volume of DMSO in E3 medium, ensuring a consistent 1:1000 (*v*/*v*) DMSO concentration across all experimental and control groups.

### 2.6 Dynamic assessment of blood glucose and insulin levels

To evaluate the stability of the T2DM model, zebrafish were washed with E3 medium post-modeling to remove residual inducing agents. Subsequently, samples from both the model and control groups were collected hourly for dynamic monitoring of blood glucose and insulin levels over a 24-h period. Blood glucose was measured as described above (*n* = 10 biological replicates, with each replicate consisting of one zebrafish larva, from three independent experiments). Insulin levels were assessed using zebrafish-specific ELISA kits (Enzyme-linked Biology, Shanghai, China), following the same protocol used for glycated hemoglobin measurement. Each experimental group included three biological replicates, with each replicate consisting of 30 zebrafish larvae.

### 2.7 Insulin tolerance test

Insulin resistance in zebrafish was assessed using an insulin tolerance test according to a previous report (Md Razip et al., 2022). At seven dpf, zebrafish embryos from both the control and model groups were administered insulin via microinjection. The insulin dose was 0.35 U/g, and injections were made at the muscle tissue located just above the swim bladder for each individual zebrafish larva. Blood glucose levels were measured at multiple time points post-injection using a glucometer to dynamically assess the insulin response. Ten biological replicates were used per group, with each replicate consisting of a single zebrafish larva.

### 2.8 Evaluation of pancreatic β-cell injury

Using the transgenic zebrafish line Tg(*ins*:EGFP) with pancreatic β-cell-specific fluorescent labeling, 10 zebrafish larvae were randomly selected from each group at the end of the experiment. Fluorescence stereomicroscope (Nikon SMZ18, Japan) was employed to capture and record images, which were then analyzed using NIS-Elements D 3.20 Advanced Imaging Software (Nikon, Japan). The fluorescence intensity of pancreatic β-cells was quantified to indirectly assess β-cell number.

### 2.9 Assessment of DN

Transgenic zebrafish lines Tg(*hb9*:EGFP) and Tg(*mpx*:EGFP) were used to evaluate DN in five dpf larvae. Following 2 d of HFHG feeding or treatment with NA (125, 250, and 500 μg/mL), 10 zebrafish larvae from each group were randomly selected for fluorescence stereomicroscope imaging. The captured images were analyzed using NIS-Elements software to quantify neuronal fluorescence intensity and the number of neutrophils in peripheral nerve regions.

### 2.10 Myelin sheath staining

FluoroMyelin™ dye, a lipophilic fluorescent probe, was employed to label myelin proteins and myelin-associated lipid-rich structures in the zebrafish model. At the end of the experimental treatment, 10 zebrafish larvae were randomly selected from each group for live myelin staining using FluoroMyelin™ Green fluorescent dye (Thermo Fisher Scientific CAT: F34651, MA, USA). Following staining, zebrafish were imaged using a fluorescence stereomicroscope, and the myelin fluorescence intensity was quantified using ImageJ software (National Institutes of Health, Bethesda, MD, USA, version 1.53k).

### 2.11 Neurobehavioral analysis

WT zebrafish at five dpf were randomly assigned to two groups: control (E3 medium) and model. After 2 d of treatment, the zebrafish were transferred to a 96-well plate, allowed to acclimate for 5 min, and then permitted to swim freely under light conditions for 15 min (*n* = 10 biological replicates, with each replicate consisting of one zebrafish larva, from three independent experiments). Total swimming distance (mm) was quantified using EthoVision XT software (version 11.5, Noldus Information Technology, Wageningen, Netherlands).

### 2.12 Fluorescent detection of ROS in zebrafish

WT zebrafish at five dpf were randomly assigned to five groups: control (E3 medium), model, and model treated with NA at concentrations of 125, 250, and 500 μg/mL. After 2 d of exposure, the zebrafish were washed twice with E3 medium. Following the manufacturer’s instructions, the zebrafish were stained with CM-H2DCFDA (Thermo Fisher Scientific CAT: C6827, MA, USA) and transferred to 96-well plates at a density of two fish per 100 μL per well (*n* = 10 biological replicates, with each replicate consisting of two zebrafish larvae, from three independent experiments). Fluorescence intensity of ROS in the zebrafish was measured using a multifunctional microplate reader at 535 nm.

### 2.13 RNA extraction and quantitative real-time polymerase chain reaction (qRT-PCR)

After 2 d of HFHG feeding or treatment with varying concentrations of NA (125, 250, and 500 μg/mL), zebrafish were immediately washed with E3 medium. Total RNA from each group was extracted using an automated nucleic acid extractor (Auto-Pure32A, Allsheng, Hangzhou, China). The concentration and purity of the extracted RNA were assessed using a UV-Vis spectrophotometer (Nanodrop 2000; Thermo Fisher, MA, USA). cDNA was synthesized from total RNA using the FastKing cDNA Synthesis Kit (Tiangen, Beijing, China). qRT-PCR was performed following the instructions provided with the SYBR qPCR Premix Kit (Vazyme, Nanjing, China). The primer sequences used in this study are listed in Table 1 *β-actin* served as the internal control, and relative mRNA expression levels were calculated using the 2^−ΔΔCt^ method. Each experimental group included three biological replicates, with each replicate consisting of 30 zebrafish larvae.

**TABLE 1:** Primers for qRT-PCR.

Gene name	Forward sequence (5′- 3′)	Reverse sequence (5′- 3′)
*mbpa*	TCT​GGA​CAA​AAC​CCC​TTC​GG	GTG​GTG​GGG​TCT​CTT​TCC​CT
*mpz*	ATC​ACA​GCA​AAA​ACA​GGC​CG	GTG​AGA​CTA​AAA​AGT​GTG​TGT​GAC​T
*il-1b*	GAA​CAG​AAT​GAA​GCA​CAT​CAA​ACC	ACG​GCA​CTG​AAT​CCA​CCA​C
*crp5*	GAC​ATT​AGA​GGC​TAC​TGA​AGG​TTA	CGC​ATG​CAG​AGA​GTA​AAC​GC
*il6*	TCA​ACT​TCT​CCA​GCG​TGA​TG	TCT​TTC​CCT​CTT​TTC​CTC​CTG
*tnf-α*	GCG​CTT​TTC​TGA​ATC​CTA​CG	TGC​CCA​GTC​TGT​CTC​CTT​CT
*β-actin*	TCG​AGC​AGG​AGA​TGG​GAA​CC	CTC​GTG​GAT​ACC​GCA​AGA​TTC

### 2.14 Metabolomics analysis

At five dpf, wild-type (WT) zebrafish were divided into three groups: control, model, and treatment (NA, 500 μg/mL) for 2 days. Following treatment, 90 zebrafish were collected per group for each biological replicate, with a total of six biological replicates. Metabolites were extracted using an organic solvent precipitation method. Each sample was homogenized in 1:9 (mg:μL) ratio of zebrafish tissue and chromatography-grade water using a Bead Ruptor Elite homogenizer (OMNI International, USA) at 30 s, 6 m/s, three cycles. 4-Chloro-phenylalanine served as the internal standard. After homogenization, 120 μL of methanol containing 200 ppb of internal standard was added, followed by vortexing for 60 s. Protein precipitation was performed at −80 °C for 2 h, and samples were centrifuged at 16,000 g for 10 min at 4 °C. The supernatant (150 μL) was transferred to a new tube, with 5 μL aliquots pooled for quality control.

Metabolomics analysis was conducted on a Thermo Scientific Vanquish UHPLC system coupled with a Thermo Scientific Orbitrap Exploris 240 mass spectrometer (USA), using both negative and positive ionization modes. Chromatographic separation was achieved using a Waters ACQUITY UPLC®HSS T3 column (100 mm × 2.1 mm, 1.8 μm) at a flow rate of 0.35 mL/min and column temperature of 40 °C. The mobile phase in positive mode consisted of 0.1% formic acid in water (A) and 0.1% formic acid in acetonitrile (B), while in negative mode, 2 mM NH_4_HCO_3_ in water (A) and acetonitrile (B) were used. The gradient was: 2% B (1 min), 50% B (2 min), 100% B (6 min), hold for 3 min, then return to 2% B in 0.1 min, and hold for 2.9 min. Mass spectrometry data was acquired over a range of 100–1000 m/z using heated electrospray ionization. Quality control was ensured by analyzing QC samples (pooled from all study samples) after every 10 test samples to maintain consistency and stability.

Mass spectrometry data were analyzed using the commercial software Progenesis QI and the R-based metabolomics package *metaX*. Metabolites were identified by referencing the kyoto encyclopedia of genes and genomes (KEGG), human metabolome database (HMDB), and LipidMaps databases. Differential metabolites were screened by integrating multivariate and univariate statistical analyses. Partial least squares discriminant analysis (PLS-DA) was first employed to calculate Variable importance in projection (VIP) scores from the first two principal components. These results were combined with fold change (FC) analysis and *p*-values from univariate testing. Metabolites were considered significantly different if they met all three criteria: VIP ≥1, |FC| ≥ 2, and corrected *p*-values (FDR <0.01). Pathway analysis of the identified differential metabolites was performed using the KEGG database.

### 2.15 Statistical analysis

All results are presented as mean ± standard deviation (SD), with *n* ≥ 3. A two-tailed *P-*value <0.05 was considered statistically significant. Statistical analyses were performed using SPSS version 22.0 (IBM Corporation, New York, USA). Metabolomics data were processed and visualized using RStudio (R version 2025.05.1 + 513). Data normality was assessed using the Shapiro-Wilk test, and homogeneity of variance was tested using Levene’s test. For comparisons between two groups, a Student’s t-test was applied. For comparisons involving more than two groups, one-way ANOVA was conducted, followed by Tukey’s post-hoc test for multiple pairwise comparisons. The effectiveness analysis was performed using PASS software version 2021 (NCSS Corporation, Utah, USA). Two key outcome variables, blood glucose levels and peripheral nerve fluorescence intensity, were selected based on the literature. A significance level (α) of 0.05 and a statistical power of 80% were applied in the calculations. Based on blood glucose data, the estimated total sample size was six implants (3 per group), while peripheral nerve fluorescence intensity data indicated that 30 implants (6 per group) would be required. Therefore, the number of zebrafish included in this study met the requirements of the power analysis and ensured sufficient statistical power to support the validity of the results.

## 3 Results

### 3.1 Establishment and validation of a zebrafish model of T2DM

This study aimed to establish a metabolically stable zebrafish model of T2DM for *in vivo* drug screening. From five to seven dpf, zebrafish were fed a HFHG (egg yolk + glucose), and a single STZ injection was administered at five dpf to induce β-cell damage. This combination of diet-induced insulin resistance and STZ-mediated β-cell cytotoxicity effectively induced T2DM (Figure 1A). At seven dpf, model validation showed significantly increased glycated hemoglobin (HbA1c; Figure 1B) and blood glucose levels (Figure 1C) compared to controls, confirming persistent hyperglycemia and successful early T2DM induction. To further assess the stability of the diabetic phenotype, we conducted a withdrawal experiment in which the HFHG feeding was discontinued. Even 24 h after withdrawal, blood glucose levels remained significantly elevated (Figure 1D), and insulin levels initially rose then declined (Figure 1E), suggesting sustained hyperglycemia and compensatory hyperinsulinemia characteristic of early-stage T2DM. Additionally, immediately following the induction period, other metabolic features were assessed. Insulin tolerance testing revealed a diminished response to exogenous insulin in the model group (Figure 1F), along with a significantly increased area under the curve (AUC) (Figure 1G), indicating systemic insulin resistance. Moreover, imaging of Tg(*ins*:EGFP) transgenic zebrafish demonstrated reduced pancreatic β-cell fluorescence in the model group (Figure 1H), with quantitative analysis confirming a significant decrease in EGFP intensity (Figure 1I), consistent with β-cell dysfunction.

Finally, we assessed the pharmacological responsiveness of the model by administering different concentrations of pioglitazone and acarbose. Pioglitazone significantly reduced blood glucose levels at all tested doses, while high-dose acarbose (150 μg/mL) also exerted a glucose-lowering effect (Figure 1J), demonstrating the model’s sensitivity to conventional antidiabetic drugs. In summary, this zebrafish model faithfully recapitulates key metabolic and pathological features of T2DM, exhibiting high stability, reproducibility, and pharmacological relevance.

### 3.2 Assessment of neuropathy in a zebrafish model of T2DM

**FIGURE 2:**
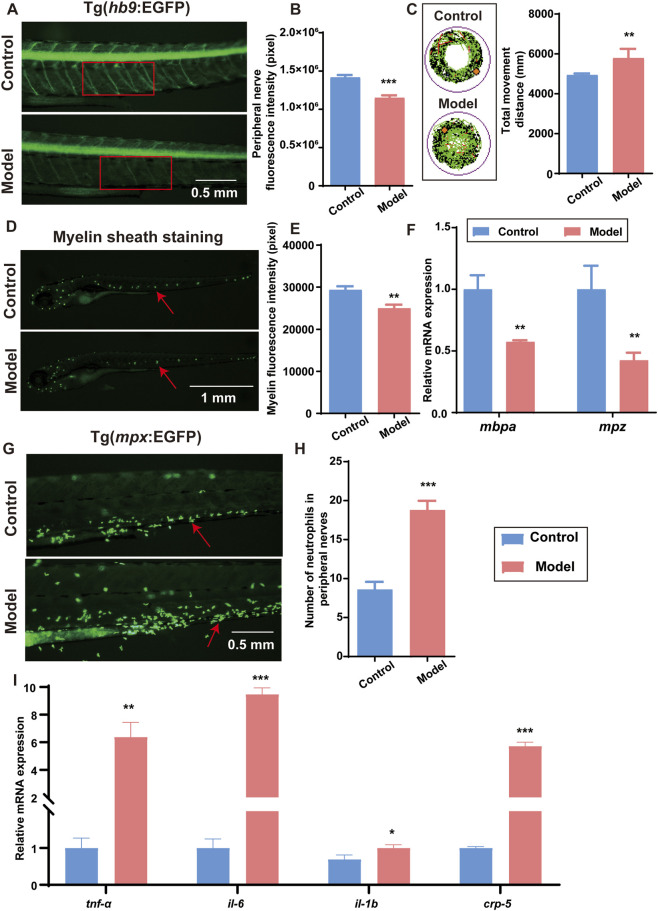
Neuropathic alterations in the diabetic zebrafish model. **(A)** Fluorescence microscopy image of motor neurons in Tg(*hb9*:EGFP) transgenic zebrafish. **(B)** Quantification of fluorescence intensity in peripheral nerves. **(C)** Impact of the diabetic model on zebrafish swimming behavior. **(D)** *In vivo* fluorescence imaging of myelin sheath staining. **(E)** Quantitative analysis of myelin fluorescence intensity. **(F)** Relative mRNA expression levels of myelin-associated genes *mbpa* and *mpz*. **(G)** Fluorescence microscopy image of neutrophils in peripheral nerves of Tg(*mpx*:EGFP) transgenic zebrafish. **(H)** Quantification of neutrophil numbers in peripheral nerves. **(I)** Relative mRNA expression levels of inflammation-related genes *tnf-α*, *il6*, *il1b*, and *crp5*. Red boxes and arrows indicate representative regions and structures selected for fluorescence quantification. Data are presented as mean ± SD. *n* = 10 biological replicates for **(A-E)** and **(G-H)**, *n* = 3 biological replicates for **(F,I)**. All data were analyzed using the Student’s t-test for two-group comparisons. *p < 0.05, **p < 0.01, ***p < 0.001 vs. control group.

To assess neural damage in the T2DM zebrafish model, Tg(*hb9*:EGFP) transgenic zebrafish were employed to visualize motor neurons. The model group showed significantly reduced EGFP fluorescence in peripheral nerves (Figures 2A,B), indicating substantial motor neuron damage under diabetic conditions. Behavioral analysis further revealed increased total swimming distance (Figure 2C), suggestive of heightened sensitivity to stimuli and escape-like hyperactivity, consistent with neural dysfunction (Shin et al., 2025). *In vivo* fluorescent myelin staining showed diminished fluorescence in the model group (Figure 2D), with quantification confirming a significant reduction in fluorescence intensity (Figure 2E). Concurrently, mRNA expression levels of myelin-associated genes *mbpa* and *mpz* were significantly downregulated in the model group (Figure 2F), further supporting the presence of structural myelin damage. Furthermore, to investigate neuroinflammatory responses, Tg(*mpx*:EGFP) transgenic zebrafish were used to label neutrophils. A pronounced increase in neutrophil infiltration around peripheral nerves was observed in the model group (Figures 2G,H), indicating inflammatory cell recruitment. Consistent with this, mRNA expression levels of inflammatory cytokines *tnf-α*, *il-6*, *il-1b*, and *crp5* were significantly elevated in the model group (Figure 2I), providing strong evidence of a robust neuroinflammatory response. In summary, the T2DM zebrafish model established in this study exhibits not only pancreatic β-cell dysfunction and sustained hyperglycemia but also features of DM, including peripheral neuronal injury, myelin abnormalities, behavioral deficits, and neuroinflammation.

### 3.3 Neuroprotective effect of NA against DN in T2DM zebrafish

**FIGURE 3:**
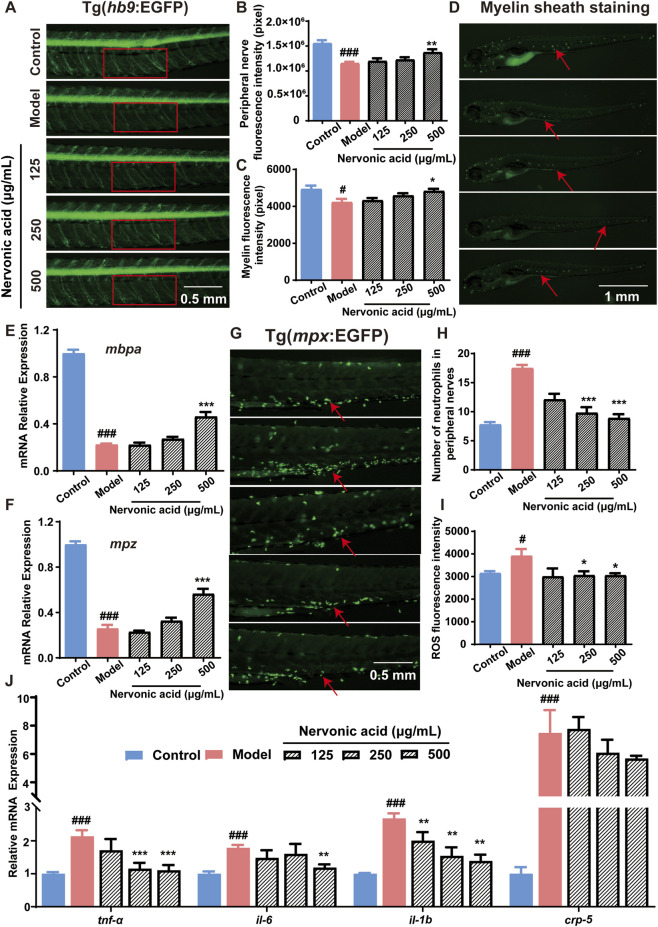
NA alleviates DN in Zebrafish by promoting nerve regeneration, enhancing myelin repair, and suppressing neuroinflammation and oxidative stress. **(A)** Representative fluorescence images of motor neurons in Tg(*hb9*:EGFP) zebrafish treated with different NA concentrations. **(B)** Quantitative analysis of EGFP fluorescence intensity in peripheral nerves of Tg(*hb9*:EGFP) zebrafish. **(C,D)** Myelin fluorescence images and corresponding intensity analysis following NA treatment. **(E,F)** mRNA expression of myelin-associated genes *mbpa* and *mpz*. **(G)** Fluorescence images of neutrophils in peripheral nerves of Tg(*mpx*:EGFP) zebrafish **(H)** Quantification of neutrophil numbers in peripheral nerves. **(I)** Quantitative analysis of whole-body ROS fluorescence intensity. **(J)** mRNA expression levels of inflammation-genes, including *tnf-α*, *il6*, *il1b*, and *crp5*. Red boxes and arrows indicate representative regions and targets used for fluorescence quantification. Data are presented as mean ± SD. *n* = 10 biological replicates for **(A-D)** and **(G-I)**, *n* = 3 biological replicates for **(E-F,J)**. All data were analyzed using one-way ANOVA followed by Tukey’s post-hoc test for multiple group comparisons. *p < 0.05, **p < 0.01, ***p < 0.001 vs. control group; ^#^ *p* < 0.05, ^###^ *p* < 0.001 vs. model group.

The protective effects of NA in diabetic neuropathy may differ depending on the dosage and disease stage at which intervention occurs. To systematically evaluate NA’s therapeutic potential, this study employed a zebrafish model of T2DM and administered NA at graded concentrations (125, 250, and 500 μg/mL). A comprehensive evaluation was conducted from multiple perspectives, including nerve regeneration, myelin repair, anti-inflammatory activity, and antioxidant capacity. In this study, motor neuron was visualized using Tg(*hb9*:EGFP) zebrafish (Figure 3A). The results revealed a significant reduction in fluorescence intensity in the diabetic model group. NA treatment restored fluorescence in a concentration-dependent manner (Figure 3B). *In vivo* myelin staining showed markedly reduced fluorescence in the model group, which was partially restored by NA (Figures 3C,D). At the molecular level, expression of the myelin-related genes *mbpa* and *mpz*, involved in myelin development and integrity, was significantly reduced in the model group but restored following treatment with NA (Figures 3E,F), supporting NA’s role in promoting myelin repair. Collectively, these findings demonstrate that NA effectively alleviates diabetes-induced neural damage in a dose-dependent manner, promoting motor neuron recovery and myelin repair in T2DM zebrafish.

Inflammation and oxidative stress are key contributors to DN. Neutrophil infiltration was visualized in Tg(*mpx*:EGFP) zebrafish (Figure 3G), showing marked accumulation in the model group. NA treatment significantly reduced neutrophil presence, especially at 500 μg/mL (Figure 3H). In parallel, mRNA analysis revealed elevated expression of proinflammatory cytokines *tnf-α*, *il-6*, *il-1b*, and *crp-5* in the model group, which was significantly suppressed by NA in a dose-dependent manner. Specifically, at a NA concentration of 250 μg/mL, suppression was statistically significant for *tnf-α* and *il-1b*. At the higher concentration of 500 μg/mL, *tnf-α*, *il-6*, and *il-1b* showed statistically significant suppression. However, *crp-5* levels were not significantly affected by NA treatment at any of the concentrations tested (Figure 3J). These findings highlight the potent anti-neuroinflammatory properties of NA, with a dose-dependent effect on cytokine suppression. Oxidative stress was evaluated via ROS staining, which showed a substantial increase in ROS levels in the model group. NA treatment markedly reduced ROS fluorescence, indicating strong antioxidant capacity, particularly at the highest dose (Figure 3I). Collectively, these findings highlight the multifaceted neuroprotective effects of NA in DN, including the promotion of nerve and myelin repair, inhibition of inflammation, and reduction of oxidative stress, ultimately supporting its potential as a therapeutic agent for the prevention and treatment of DN.

### 3.4 Metabolomic analysis reveals the protective mechanism of NA in DN

**FIGURE 4:**
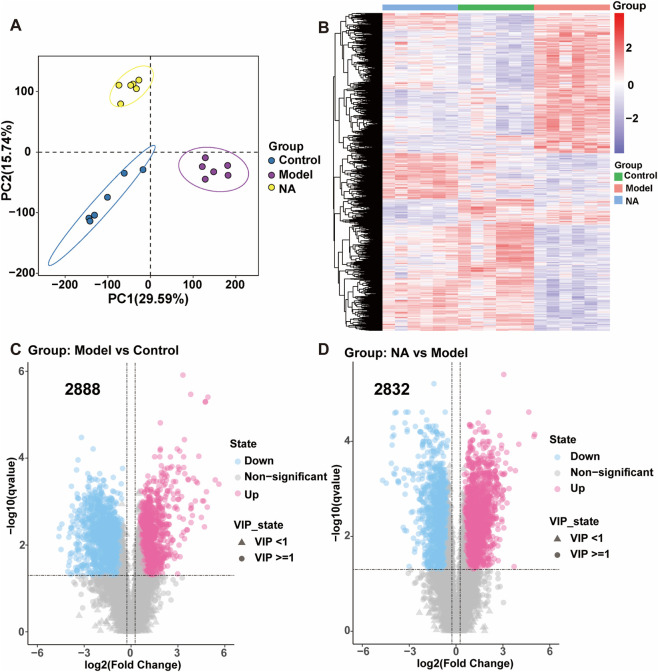
Metabolomic analysis of zebrafish. **(A)** PLS-DA score plot comparing the control group, model group, and nervonic acid treatment group (500 μg/mL). **(B)** Heatmap showing differential metabolite expression among the groups. Volcano plot of the metabolome, **(C)** model vs. control groups and **(D)** NA vs. model. Corrected *p* (FDR) < 0.01, fold change (FC) > 2 and VIP≥1. *n* = 6 biological replicates, each replicate contains 90 zebrafish larvae.

Diabetes is a complex metabolic disorder characterized by widespread alterations in small-molecule metabolites. Metabolomics is widely applied to detect such metabolic changes under different treatment conditions and can help identify potential therapeutic targets. In this study, a non-targeted metabolomics approach was employed to explore the metabolic mechanisms underlying the protective effects of NA on DN and to identify key intervention-related metabolites and pathways. PLS-DA revealed clear separation among the control, model, and NA-treated groups, with principal components PC1 and PC2 jointly explaining 45.33% of the total metabolic variance (Figure 4A), indicating distinct metabolic profiles among groups. Heatmap analysis further demonstrated that NA treatment markedly reversed the metabolic disturbances observed in the model group, shifting the overall metabolic pattern toward that of the control group (Figure 4B), indicating that NA effectively restores metabolic homeostasis. To identify key metabolites, pairwise comparisons were performed between the Model vs. Control and NA vs. Model groups. Volcano plot analysis revealed 2,888 significantly altered metabolites between the Model and Control groups (Figure 4C), and 2,832 differential metabolites between the NA and Model groups (Figure 4D), highlighting the extensive metabolic changes induced by diabetes and the substantial modulatory effects of NA.

**FIGURE 5:**
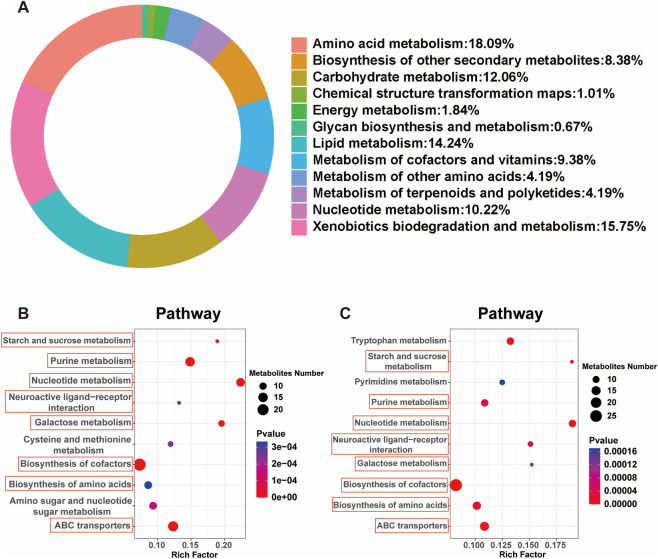
KEGG enrichment reveals NA-regulated metabolic and signaling pathways in DN. **(A)** KEGG Pathway Functional Classification Ring Diagram. **(B)** KEGG pathway enrichment analysis between the Model and Control groups. **(C)** KEGG pathway enrichment analysis between the NA and Model groups.

Kyoto encyclopedia of genes and genomes (KEGG) pathway enrichment analysis revealed that the annotated metabolites were primarily involved in amino acid metabolism (18.09%), lipid metabolism (14.24%), carbohydrate metabolism (12.06%), and nucleotide metabolism (10.22%) (Figure 5A). Additionally, significant enrichment was observed in pathways related to xenobiotic biodegradation (15.75%) and the metabolism of cofactors and vitamins (9.38%). These results suggest that the differential metabolites are largely associated with core metabolic functions and stress responses, implying disruptions in energy homeostasis, membrane composition, and detoxification processes under diabetic conditions. Further KEGG enrichment analysis identified eight shared pathways between the model vs. control and NA vs. model comparisons (Figures 5B,C). These included starch and sucrose metabolism, purine metabolism, neuroactive ligand-receptor interaction, and ABC transporters, all of which are closely linked to energy metabolism, neural signaling, and transmembrane transport. These overlapping pathways highlight the central role of metabolic and signaling disturbances in DN and suggest that NA may exert its protective effects by modulating these key biological processes.

**FIGURE 6:**
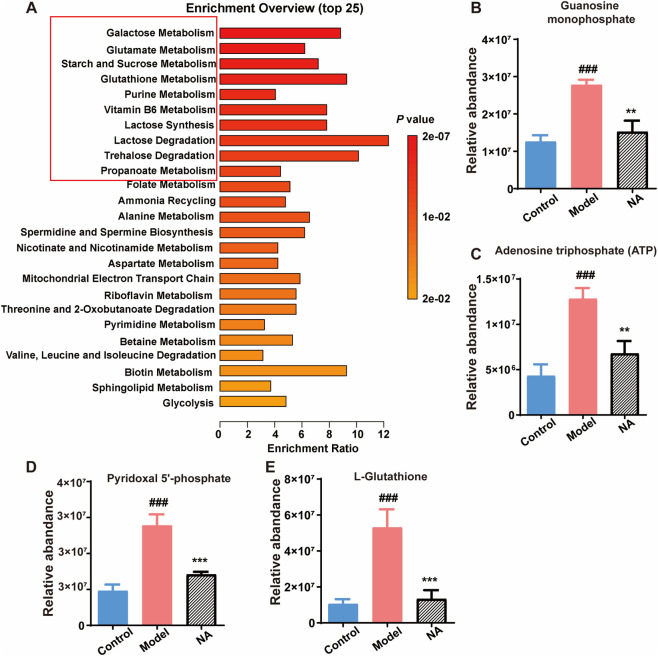
NA reverses diabetes-induced metabolic disturbances in key pathways associated with DN. **(A)** KEGG pathway enrichment analysis of metabolites involved in the eight pathways commonly identified in both model vs. control and NA vs. model comparisons. **(B–E)** Relative abundance of GMP, ATP, PLP, and L-glutathione in the Control, Model, and NA-treated groups. Panels **(B–E)** were analyzed using one-way ANOVA followed by Tukey’s post-hoc test for multiple comparisons. **p < 0.01, ***p < 0.001 vs. control group; ^###^ *p* < 0.001 vs. model group.

To gain deeper insight into the mechanisms underlying NA’s protective effects, we further analyzed the shared metabolic pathways identified in both the model vs. control and NA vs. model comparisons. KEGG enrichment analysis was performed on the overlapping metabolites involved in these eight common pathways (Figure 6A). Among the top 10 enriched pathways, four metabolites closely linked to the pathophysiology of diabetic neuropathy were identified: guanosine monophosphate (GMP), adenosine triphosphate (ATP), pyridoxal 5′-phosphate (PLP), and L-glutathione. These metabolites were significantly upregulated in the model group but markedly reduced following NA treatment (Figures 6B–E), indicating that NA effectively reverses DN-associated metabolic disruptions.

## 4 Discussion

DN is one of the most common and debilitating complications of T2DM, characterized by progressive peripheral nerve damage, myelin disruption, and neuroinflammation (Callaghan et al., 2012). Despite the increasing global prevalence of T2DM, current clinical options for preventing or reversing DN remain limited and are often associated with inadequate efficacy or adverse effects (Elafros et al., 2022). As a result, increasing attention has been directed toward naturally derived bioactive compounds with neuroprotective and anti-inflammatory properties (Xu et al., 2024). Among these, long-chain monounsaturated fatty acids such as NA have shown promising therapeutic potential in various neurological disorders (Wang et al., 2023). NA is a very long-chain monounsaturated fatty acid that plays a crucial role in myelin biosynthesis and the maintenance of neural integrity, making it a compelling candidate for interventions targeting demyelinating and neurodegenerative conditions (Zheng et al., 2024). In the present study, we successfully established a metabolically stable zebrafish model of T2DM that reproduces key pathological features of DN, including motor neuron impairment, myelin disruption, behavioral hyperactivity, and neuroinflammation. Using this model, we systematically evaluated the neuroprotective effects of NA. Our findings demonstrate that NA confers multifaceted protective effects against T2DM-induced DN. Specifically, NA ameliorated diabetes-induced motor neuron damage and myelin abnormalities, reduced peripheral neutrophil infiltration, suppressed the expression of proinflammatory cytokines, and enhanced antioxidant defenses. These results provide novel mechanistic insights into the therapeutic potential of NA for DN. As far as we know, our work is the first report of animal research on the application of NA to the treatment of DN.

In this study, we established a T2DM model in zebrafish larvae by combining a high- HFHG diet with a single low-dose STZ injection at five dpf. Unlike previous zebrafish models that relied on morpholino knockdown (Zang et al., 2019), transgenic manipulation (Sharchil et al., 2022), or direct STZ injection(Cao et al., 2023), the HFHG + STZ method offers a minimally invasive, physiologically relevant alternative with advantages in speed, reproducibility, and scalability. Although HFHG + STZ has been used for long-term modeling in mammals (Magalhães et al., 2019), our approach induces stable hyperglycemia, insulin resistance, and β-cell dysfunction within just 2 days, significantly shortening the induction period compared to mammalian models. Additionally, the model responded pharmacologically to standard antidiabetic drugs, such as pioglitazone and acarbose, confirming its translational relevance and utility for *in vivo* drug screening.

Although glucose control is central to diabetes management, it alone is insufficient to prevent the onset or progression of DN, as patients with well-regulated blood glucose levels may still develop or experience worsening neuropathic symptoms (Feldman et al., 2019). DN is a multifactorial complication involving hyperglycemia-induced intracellular stress, dyslipidemia, oxidative damage, immune dysregulation, and chronic inflammation (Yang et al., 2025; Arı et al., 2025). Proinflammatory cytokines, including TNF-α, IL-1β and IL-6, are known to exacerbate DN progression, and their inhibition has been shown to alleviate disease severity (Nashtahosseini et al., 2025). Furthermore, DN is associated with impaired nerve conduction, altered Na^+^/K^+^-ATPase activity, and excessive ROS production, all of which contribute to neuronal injury and demyelination (Eid et al., 2023). In this study, NA administration significantly alleviated the pathological features of DN in diabetic zebrafish. Structurally, NA restored motor neuron integrity and myelination, as evidenced by enhanced fluorescence signals and upregulated expression of myelin-associated genes (*mbpa*, *mpz*). However, it is important to note that FluoroMyelin is not a specific marker for myelin proteins and may also label other lipid-rich structures, such as mechanosensory cells. Therefore, we acknowledge the limitations of FluoroMyelin as a sole marker for myelin. To further assess its effects on myelin, we complemented the structural observations with gene expression analysis of myelin-associated biomarkers. The *mbpa* gene encodes myelin basic protein (MBP), a critical structural component of the myelin sheath that facilitates tight compaction of myelin layers and ensures efficient nerve impulse conduction (Cruz-Méndez et al., 2022). The *mpz* gene encodes myelin protein zero, a major transmembrane glycoprotein essential for the formation and maintenance of myelin in the peripheral nervous system, contributing to membrane adhesion and structural stability (Shy et al., 2004). The observed upregulation of both genes following NA treatment suggests a direct role in promoting remyelination and restoring axonal function under diabetic conditions. In addition to structural repair, NA exerted potent anti-inflammatory and antioxidative effects. It reduced neutrophil infiltration and downregulated proinflammatory cytokines (*tnf-α*, *il-6*, *il-1β*, *crp5*), while also suppressing ROS accumulation. These actions likely reflect improved mitochondrial function or enhanced free radical scavenging, contributing to the preservation of neural tissue (Li et al., 2025). Therefore, our results suggest that NA mitigates DN through both structural and biochemical mechanisms.

In the metabolomic analysis of T2DM-induced DN in zebrafish larvae, the differential metabolites were predominantly enriched in pathways related to amino acid, purine and lactose metabolism. These pathways are essential for maintaining neural function, redox homeostasis, and cellular energy balance, which are frequently disrupted under diabetic conditions (Handzlik et al., 2023; Yao et al., 2023). KEGG pathway enrichment revealed that NA effectively attenuated the dysregulation of several key metabolites associated with DN, particularly GMP, ATP, PLP, and L-glutathione. These metabolites are closely linked to neural function, redox homeostasis, and energy metabolism, which are commonly disrupted in diabetes. Purine metabolism plays a central role in regulating cellular signaling and energy transfer (Huang et al., 2021). In diabetic models, elevated GMP levels are linked to metabolic stress and disrupted guanine nucleotide cycling, which can impair axonal transport and neurotrophic signaling (Wang et al., 2011). NA-mediated normalization of GMP levels suggests a restoration of purine metabolism and neuronal cellular homeostasis. Similarly, ATP is a critical molecule involved in cellular energy metabolism (Cojocaru et al., 2023), paradoxically increases in hyperglycemic conditions due to mitochondrial overactivation and compensatory metabolic flux, preceding mitochondrial dysfunction and ROS accumulation (Pinti et al., 2019; Cojocaru et al., 2023). The reduction of ATP levels following NA treatment likely indicates a decrease in metabolic overload and an improvement in mitochondrial efficiency. In our study, we observed elevated ATP and GMP levels in the diabetic model, indicating metabolic dysregulation. The compensatory elevation of ATP in the early stages of high-glucose exposure is a well-documented phenomenon, where mitochondria temporarily enhance glucose oxidation, leading to transient increases in ATP production. However, this compensatory increase in ATP does not signify improved energy metabolism but rather reflects a metabolic imbalance. This imbalance is often characterized by excessive ROS generation and accelerated purine degradation (Zhao et al., 2018; Chareyron et al., 2020). Purine metabolism disturbances, particularly the accumulation of GMP, are hallmarks of diabetic neuropathy. The abnormal accumulation of intermediates such as GMP and GTP exacerbates oxidative stress, increases uric acid levels, and activates inflammation-related pathways, such as the NLRP3 inflammasome. These disturbances contribute to sustained damage to Schwann cells and the axonal microenvironment, which are crucial for myelin formation (Calcutt, 2020; Patritti-Cram et al., 2021). PLP, the bioactive form of vitamin B6, functions as a coenzyme in neurotransmitter biosynthesis and amino acid metabolism (Rivero et al., 2024). Imbalances in PLP may impair synaptic transmission and increase the risk of neurotoxicity (Kasaragod et al., 2020). Its downregulation post-NA treatment may reflect rebalanced vitamin B6 metabolism, reducing potential neurotoxicity and supporting neural health. L-glutathione, a key intracellular antioxidant, plays a central role in mitigating ROS-mediated damage (Kwon et al., 2019). Elevated glutathione levels in diabetic conditions likely represent a compensatory response to oxidative stress, and NA’s ability to normalize these levels suggests reduced oxidative burden and enhanced redox balance.

We hypothesize that the normalization of ATP and GMP levels by NA contributes to the improvement of Schwann cell function and myelin repair. Schwann cells, which are highly sensitive to metabolic and energy states during myelination, rely on purine metabolism for lipid synthesis, myelin protein expression, and regulation of small GTPases—all crucial for the formation and maintenance of myelin (Montani et al., 2018; Patritti-Cram et al., 2021). Moreover, the correction of purine metabolism by NA may reduce the production of inflammatory mediators, aligning with the observed reduction in inflammation (Zhao et al., 2018). This mechanism helps explain the phenotypic improvements observed in our model, including myelin repair and reduced inflammation.

While these findings highlight the neuroprotective and metabolic effects of NA in DN, several limitations must be considered. The zebrafish model, while useful due to its neurodegenerative similarities with mammals, has significant differences in physiology and immunology, meaning that the results may not fully translate to humans. Additionally, zebrafish have a higher capacity for myelination and neuronal regeneration, which may exaggerate NA’s effects compared to mammals. The short 2-day treatment duration limits our understanding of NA’s long-term potential in sustaining myelin repair and behavioral improvements. Moreover, the dose range of 125–500 μg/mL lacks a clear biological or clinical rationale, and the pharmacokinetics of NA in zebrafish remain unknown, making the exposure-dose-effect relationship unclear. Finally, while we observed metabolic changes such as elevated ATP and GMP, the causal links to myelin repair are not fully established, and further studies using targeted metabolomics or proteomics are needed to explore the underlying molecular mechanisms of NA’s effects.

## 5 Conclusion

Given the limitations of current therapeutic options for DN, there is an urgent need for safer and more effective neuroprotective strategies. This study demonstrates that NA, a naturally occurring long-chain monounsaturated fatty acid, exerts significant protective effects against DN by promoting neural repair, reducing inflammation and oxidative stress, and restoring metabolic balance. As a bioactive compound derived from natural sources, NA holds strong potential for development into functional interventions for DN prevention and treatment.

## Data Availability

The original contributions presented in the study are included in the article/supplementary material, further inquiries can be directed to the corresponding authors.
